# The Value of Dental Legislation

**Published:** 1904-12

**Authors:** 

**Affiliations:** Batavia, Java.


### THE VALUE OF DENTAL LEGISLATION.

By Dr. Anema, Batavia, Java.

(Abstract of paper read before the Fourth International Dental Congress, St. Louis, 1904.)

The interests of one individual are often opposed to the interests of another. The interests of a number of individuals belonging to the same class can, to some extent, be opposed to the interests of the rest of men. According to the well recognized principle, "The greatest good to the greatest number," the interest of the class must be subordinate to that of the mass, i. e., the general public; yet it has sometimes happened that the power of a class was so well organized that for a long time the interests of the minority predominated those of the majority.

We find this condition where a certain class has been long enough in power to make the laws of the country.

Allow me to ask you the following question: What do you consider the true value of dental legislation and what may the public expect from it?

In the case before us of the public interest vs. dental legislation, allow me to introduce as the principal factor making for ill "professional egotism," a spirit related to self-preservation of the class on one side and professional jealousies on the other. Here I must diverge a moment to make myself better understood.

To my mind there is no more doubt of the existence of professional egotism than there is of a certain kind of patriotism, called "jingoism," that preaches, "My country, right or wrong," or of that egotism which, by instinct and brute force, extinguishes weaker races. Of the latter we have in the history of mankind many instances. Of the former, I hope to give you an instance later on.

All these forms of egotism are brought about by instincts of the individual becoming active in the mass at certain times and intervals of its existence.

Among the strongest instincts of man is the one of self-preservation. It is a common truth that instincts are hard to deal with, if indeed they can be dealt with at all. They may become concealed to the inexperienced eye, covered by today's civilization, which often times is not much more than a social veneer, or by an amiable self-deceit of good-natured and well-to-do-people who believe that at least among their classes, instincts, and especially the one of self-preservation, are rudimentary. If, however, those less critical believe that the better classes look out mainly for the interests of others, then to my mind they are mistaken. The lesson given nearly two thousand years ago, "Love thy neighbor as thou lovest thyself," is still generally applicable, which implies its reverse that as a rule man looks out more for his own interests than for the interests of others. I will say, however, that there are no exceptions to this rule, such people as martyrs, where the instinct of self-preservation seems almost lost, but this specimen is very rare. This exception shows up more clearly the rule that says: "The instinct of self-preservation is stronger than the instinct of altruism."

When a man joins a profession he brings his instinct of self-preservation with him. This brings him often in closer contact with the profession, as he hopes that the profession may at a time be of some use to him.

The following is a direct proof of the existence of such an instinct. In a certain country a society of dentists, forming the editorial staff of a dental journal, desired to change the rules. One of the members proposed the following as the principle on which the new structure should be erected: "The society intends to serve the public by promoting dentistry in the most remote sense of art and science." His obvious reason, as he explained at the time, was to have as the leading motive the "interest of the public." After having been carefully considered, the proposition was rejected almost unanimously. So far as the thoughts of the opposition could be understood, the men who voted against the public interest proposition, feeling themselves representatives of their profession, considered it their duty not to look out, at least, not in the first place, for interests other than those of the body of men represented. The new rules are now based upon a foundation which can easily be laid bare and understood by looking upon the flag and emblem of some of our best dental journals which says, "devoted to the interests of the profession."

As dental journals are leaders and at the same time the voice of the profession, I consider these facts as valuable ones and as a proof that the dental profession needs devotion to its own interests. And as devotion to one (the profession) excludes devotion to another (the public) otherwise it is no devotion—what else can be the cause of the demand of this highest form of affection than the instinctive self-preservation of the class named, professional self-preservation.

In France a body of over two hundred and fifty dentists, the *"Syndicat des Chirurgiens-Dentists de France,"* use power to bring before court respectable foreign dentists for using legally acquired but foreign degrees of doctor. As their country does not furnish a dental doctor's degree, the dentists claim that the foreigners gain an undue reputation by the use of that title, thus doing harm to the interests of the local dentists.

That this feeling is more or less common to all lands was illustrated in a conversation bearing on educational matters I lately had with a dental professor of Europe. In replying to a doubt expressed by me as to the advisability of the bachelor's degree and the official medical studies, and the detriment they might work in debarring many from entering the school and in denying the blessings of dentistry to the masses, he said, jokingly, "Oh, we don't need any more dentists here."

After this I hope to be understood when I repeat professional egotism is an ill-natured feeling on one side to the self-preservation of the class and on the other to professional jealousy.—Items of Interest.

